# Anger and confrontation during the COVID-19 pandemic: a national cross-sectional survey in the UK

**DOI:** 10.1177/0141076820962068

**Published:** 2020-10-28

**Authors:** Louise E Smith, Bobby Duffy, Vivienne Moxham-Hall, Lucy Strang, Simon Wessely, G James Rubin

**Affiliations:** 1 Institute of Psychiatry, Psychology & Neuroscience, King’s College London, London SE5 9RJ, UK; 2 Faculty of Social Science & Public Policy, King’s College London, London WC2B 2BG, UK

**Keywords:** conflict, tension, Government measures, lockdown, community, corrosion

## Abstract

**Objectives:**

To investigate factors associated with anger or confronting others due to COVID-19.

**Design:**

Online cross-sectional survey.

**Setting:**

Data were collected between 17 and 20 July 2020.

**Participants:**

A total of 2237 participants living in the UK aged 16–75 years.

**Main outcome measures:**

Reporting having had arguments, felt angry or fallen out with others because of COVID-19. Reporting having confronted or reported someone to the authorities, or that you had been confronted or reported to the authorities, for not wearing a face covering; not keeping your distance from others or being in too large a group; or alternatively following recommended measures too carefully. We used logistic regression analyses to identify factors associated with anger and confrontation.

**Results:**

Most participants reported having had arguments, feeling angry or fallen out with others because of COVID-19 (56%, n = 1255). Twenty-two percent (n = 500) of participants reported that they had confronted or reported someone. Fourteen percent (n = 304) of participants reported that they had been confronted or reported by someone. Confronting someone, having been confronted and feeling angry or having had arguments were strongly associated with each other. Anger and confrontation were associated with younger age, greater likelihood of experiencing significant financial difficulties due to the pandemic, greater perceived risk of COVID-19 and getting information about COVID-19 from social media.

**Conclusions:**

Measures put in place to prevent the spread of COVID-19 have caused considerable strain. Increased support, clear messaging on the rationale for easing restrictions and combatting misinformation on social media may all help decrease tension.

## Introduction

The emergence of COVID-19 has brought with it much uncertainty. The UK Government has brought in measures to prevent the spread of COVID-19, including physical distancing measures (known colloquially as ‘social distancing’) and mandating the use of face coverings on public transport and in shops.^1^ It is unclear how long restrictions will continue, what the long-terms risks of COVID-19 are and how severe economic and social disruption will be. Evidence from previous emergencies has shown that with prolonged risk and uncertainty about the level of risk comes the potential for conflict within the community.^2,3^ It has been suggested that ‘therapeutic communities’, which are characterised by high levels of cohesion and mutual aid, are more likely to follow natural disasters, while ‘corrosive communities’, which are divided and see conflict, are more likely after human-made disasters.^3^ Pandemics are unique, because measures to control the spread of infection generate as much, and perhaps in the long term, more, impact than the disease itself. Systemic differences in the influence of the incident on different groups within the population can also cause further divide.^4^ Historically, pandemics have increased inequalities and either created or exacerbated community tensions, and it already seems unlikely that COVID-19 is the exception to this rule.^5^

Research suggests that three main inter-related factors contribute to the emergence and persistence of corrosive communities: the mental and physical wellbeing of individuals within the community; perceptions of the failure of Government and other institutions to properly uphold and execute their roles and responsibilities; and continued litigation.^3,6^ In the UK, there is convincing evidence of a true increase in the prevalence of anxiety and depression in the population compared to the pre-pandemic period.^7,8^ Confidence in the Government’s ability to handle the COVID-19 pandemic has decreased during the pandemic period (this is particularly true for England).^9^ Tension may also be influenced by disagreements about the levels of risk posed by COVID-19^2^ and non-adherence to protective behaviours. Insufficient information and misinformation is also associated with deterioration of social support.^11^ In the context of COVID-19, lower knowledge and endorsement of conspiracy theories,^12^ and poor understanding of Government measures,^10^ may be associated with anger and conflict. Information sources, such as social media, which spread misinformation widely and rapidly^13,14^ may propagate misinformation about COVID-19. Stigmatising attitudes have also been associated with decreased community resilience following an emergency.^15^

In this study, we investigated factors associated with anger and confrontation during the COVID-19 pandemic in a demographically representative sample of the UK population.

## Method

### Design

The survey was designed by researchers from King’s College London and an expert advisory group. The market research company Ipsos MORI was commissioned to carry out this cross-sectional survey between 17 and 19 July 2020.

### Participants

Participants (n = 2237; aim 2200) were recruited from Ipsos MORI’s online research panel (n = 252,000+) and were eligible if they were aged 16–75 years and were UK residents. Quota sampling was used, based on age, gender, working status and Government Office Region and balanced on social grade, to ensure that the sample was broadly representative of the UK general population. Approximately 40,000 were sent a link to take part in the survey; 89% did not access the link. Seventeen percent of those who accessed the link did not finish the survey; some of those who accessed the link were excluded as quota targets for the survey had been filled. This ‘response rate’ is typical for non-probability quota samples of this nature. Participants were screened out due to a lack of data for sociodemographic variables (post code, n = 203) or suspiciously fast completion of the survey or providing identical answers to multiple consecutive questions (n = 50). We have flagged the assumptions relating to the method we have adopted and guided readers to a useful summary in the limitations section.^16^ Upon completion of the survey, participants were entered into a prize draw.

This paper reports data from the third wave of polling run by the group; we have previously reported data from this survey wave relating to other outcomes.^17,18^

### Study materials

Full survey items are presented in the supplementary materials.

#### Anger

Participants were asked if they have had arguments with friends or family members about how to behave during the COVID-19 pandemic; if they were no longer speaking to a friend or family member because of disagreements about COVID-19; if they had felt angry with other people they know because of how they were behaving with regard to the pandemic; and if they thought other people they knew had been angry at them because of how they were behaving in relation to the COVID-19 pandemic.

#### Confrontation

We asked participants if they had confronted or reported someone to the authorities for not wearing a face mask, for not staying a sufficient distance away from others or for being in too large a group, and for following the recommended measures too carefully. We also asked participants if they had been confronted by someone for the same reasons.

#### Self-reported protective behaviours

Participants were asked whether they had worn a face mask in the last two weeks and if they had stayed 2 m away from other people when outside their home.

#### Beliefs and knowledge about COVID-19

We asked participants if they had had COVID-19. Participants were asked to what extent they thought COVID-19 posed a risk to themselves personally and people in the UK, and what they thought was their personal chance of catching the coronavirus was in the next month.

Stigmatising attitudes were measured by asking participants if they thought it was best to avoid people who had recovered from coronavirus. Participants rated their knowledge about COVID-19. Objective knowledge was measured by asking participants what they thought the most common symptoms of COVID-19 were.^19^

We asked participants if they thought the Government only wants people to wear face masks as a way of controlling them.

#### Trust in UK Government and COVID-19 measures

Participants indicated to what extent they trusted the UK Government to control the spread of coronavirus.

We asked participants if they thought the UK Government was relaxing measures to control coronavirus too quickly, too slowly or at about the right pace. Participants also rated how worried they were about the restrictions being lifted.

#### Psychological factors

Participants were asked if they had felt more anxious and depressed than normal and rated to what extent COVID-19 was stressful to them.

#### Information sources

We asked participants how much of what they knew about COVID-19 came from TV and radio broadcasters, newspapers and magazines, YouTube, Facebook, WhatsApp, Twitter, and family or friends.

#### Sociodemographic characteristics

Participants were asked for their age, gender, presence of dependent children in the household, employment status, highest educational or professional qualification, total household income, household size, marital status, ethnicity and region. We also collected socioeconomic grade. Participants were also asked how likely it was that they would face significant financial difficulties as a result of the disruption from coronavirus.

### Ethics

Ethical approval for this study was granted by the King’s College London Research Ethics Committee (reference: MRA-19/20-18251).

### Statistical reliability

A sample size of 2237 allows a 95% confidence interval of plus or minus 2 percentage points for the prevalence estimate for each survey item.

### Analysis

Details of how we recoded variables are in the supplementary materials.

For each set of analyses, we conducted univariable and multivariable regressions. Multivariable analyses controlled for sociodemographic variables (region, age, gender, presence of dependent children in the household, employment status, highest educational or professional qualification, total household income, socioeconomic grade, ethnicity, marital status and household size).

We used logistic regression analyses to investigate associations between anger and someone else being angry at you, confrontation, self-reported protective behaviours, beliefs and knowledge about COVID-19, trust in UK Government and COVID-19 measures, psychological factors, information sources and socio-demographic characteristics.

We used logistic regression analyses to investigate associations between confronting or reporting someone and anger, self-reported protective behaviours, beliefs and knowledge about COVID-19, trust in UK Government and COVID-19 measures, psychological factors, information sources and sociodemographic characteristics.

We ran logistic regression analyses investigating sociodemographic factors associated with having been confronted by someone because of COVID-19. We then investigated the influence of having been confronted (independent variable) on anger, stigma, psychological factors and trust in UK Government (dependent variables). Where the dependent variable was binary (anger, stigma and feeling more anxious and depressed), we used logistic regressions; where it was continuous (COVID-19 stress and trust in UK Government), we used linear regressions. For all analyses investigating having been confronted, we also controlled for self-reported protective behaviour.

#### Sensitivity analyses

As multiple analyses were run on single outcomes (anger, n = 37; confronting/reporting someone, n = 37), we applied a Bonferroni correction to results (*p* ≤ 0.001). Analyses that reached this significance level are marked in tables by a double asterisk (**).

## Results

### Anger

Most participants (n = 1255, 56.1%, 95% confidence interval: 54.0–58.2) reported having had arguments, feeling angry or having fallen out with someone because of the COVID-19 pandemic.

Having had arguments, feeling angry or having fallen out with someone because of the COVID-19 pandemic was associated with younger age, and thinking you were more likely to experience significant financial difficulty as a result of COVID-19 (Table 1). Also associated with anger were thinking that other people had felt angry at you; confronting/reporting someone; having been confronted or reported by someone; feeling more anxious or depressed than usual; thinking that measures put in place to prevent the spread of COVID-19 were being relaxed too quickly; having worn a face mask in the last two weeks; greater perceived risk of COVID-19 (to oneself and people in the UK); greater perceived likelihood of catching COVID-19 in the next month; perceiving greater stress of COVID-19; being more worried about restrictions being lifted; lower trust in UK Government to control the spread of COVID-19; and greater use of Facebook, WhatsApp, Twitter and YouTube as information sources about COVID-19 (Table 2).

**Table 1.** Associations between socio-demographic characteristics of participants and having had arguments, felt angry with, or having fallen out with others because of the COVID-19 pandemic.

		Univariable analyses			Multivariable analyses	
Participant characteristics	Level	Not had arguments or fallen out/felt angry with others because of the COVID-19 pandemic (n = 816)	Had arguments or fallen out/felt angry with others because of the COVID-19 pandemic (n = 1255)	Odds ratio (95% CI)	Total n	Adjusted odds ratio (95% CI)^a^
Gender	Male	419 (41.3)	596 (58.7)	Reference	1848	Reference
	Female	386 (37.4)	647 (62.6)	1.18 (0.99 to 1.41)		1.17 (0.96 to 1.42)
Age	16 to 24 years	77 (26.5)	214 (73.5)	Reference	1848	Reference
	25 to 34 years	112 (30.0)	261 (70.0)	0.84 (0.60 to 1.18)		0.68 (0.47 to 1.00)*
	35 to 44 years	12 (34.7)	228 (65.3)	0.68 (0.48 to 0.95)*		0.58 (0.39 to 0.87)*
	45 to 54 years	172 (41.5)	242 (58.5)	0.51 (0.37 to 0.70)**		0.43 (0.29 to 0.63)**
	55 years and over	334 (51.9)	310 (48.1)	0.33 (0.25 to 0.45)**		0.27 (0.19 to 0.40)**
Child in the household	None	640 (41.9)	887 (58.1)	Reference	1848	Reference
	Child present	176 (32.4)	368 (67.6)	1.51 (1.23 to 1.85)*		1.08 (0.81 to 1.45)
Employment status	Working	491 (37.4)	823 (62.6)	Reference	1848	Reference
	Not working	325 (42.9)	432 (57.1)	0.79 (0.66 to 0.95)*		0.84 (0.68 to 1.04)
Highest educational or professional qualification	Degree or higher (Bachelors, Masters, PhD)	328 (36.9)	560 (63.1)	Reference	1848	Reference
	GCSE/vocational/A-level/No formal qualifications	488 (41.3)	695 (58.7)	0.83 (0.70 to 1.00)*		0.94 (0.76 to 1.16)
Total annual household income	Up to £34,999	467 (41.1)	670 (58.9)	Reference	1848	Reference
	£35,000 or over	275 (36.5)	478 (63.5)	1.21 (1.00 to 1.46)*		0.89 (0.71 to 1.11)
Socio-economic grade	ABC1	517 (38.5)	827 (61.5)	Reference	1848	Reference
	C2DE	299 (41.1)	428 (58.9)	0.89 (0.74 to 1.08)		0.98 (0.79 to 1.22)
Household size	One	211 (47.0)	238 (53.0)	Reference	1848	Reference
	Two	306 (41.5)	432 (58.5)	1.25 (0.99 to 1.59)		0.96 (0.69 to 1.34)
	Three or more	299 (33.8)	585 (66.2)	1.73 (1.38 to 2.19)**		1.02 (0.71 to 1.45)
Marital status	Married/living as married	434 (37.8)	714 (62.2)	Reference	1848	Reference
	Single/separated/ divorced/ widowed	382 (41.4)	541 (58.6)	0.86 (0.72 to 1.03)		0.70 (0.53 to 0.91)*
Ethnicity	White	739 (40.1)	1105 (59.9)	Reference	1848	Reference
	Black and minority ethnicity	65 (33.2)	131 (66.8)	1.35 (0.99 to 1.84)		0.89 (0.62 to 1.28)
Region	North	191 (39.4)	294 (60.6)	Reference	1848	Reference
	Midlands	203 (39.4)	312 (60.6)	1.00 (0.77 to 1.29)		1.04 (0.79 to 1.38)
	South	186 (40.3)	275 (59.7)	0.96 (0.74 to 1.25)		0.98 (0.74 to 1.30)
	London	111 (39.9)	167 (60.1)	0.98 (0.72 to 1.32)		1.11 (0.79 to 1.56)
	Wales	37 (38.1)	60 (61.9)	1.05 (0.67 to 1.65)		1.13 (0.70 to 1.83)
	Scotland	68 (37.6)	113 (62.4)	1.08 (0.76 to 1.53)		1.13 (0.77 to 1.66)
	Northern Ireland	20 (37.0)	34 (63.0)	1.10 (0.62 to 1.98)		0.99 (0.54 to 1.84)
Significant financial difficulty	5-point scale, 1 = not at all likely to 5 = certain/ already am	N = 716, M = 2.04, SD = 1.05	N = 1135, M = 2.53, SD = 1.22	1.46 (1.34 to 1.60)**	1676	1.37 (1.24 to 1.52)**

For non-binary independent variables, the presented odds ratios and adjusted odds ratios relate to a one-unit increase in the factor scale.

a Adjusting for gender, age, presence of dependent child in the household, employment status, highest educational or professional qualification, total annual household income, socioeconomic grade, household size, marital status, ethnicity and region.

* *p* ≤ 0.05.

** *p* ≤ 0.001.

**Table 2.** Associations between conflict, protective behaviours, beliefs and knowledge about COVID-19, trust in UK Government and COVID-19 measures, psychological factors, information sources and having had arguments, felt angry with, or having fallen out with others because of the COVID-19 pandemic.

		Univariable analyses			Multivariable analyses	
Factor	Level	Not had arguments or fallen out/felt angry with others because of the COVID-19 pandemic (n = 816)	Had arguments or fallen out/felt angry with others because of the COVID-19 pandemic (n = 1255)	Odds ratio (95% CI)	Total n	Adjusted odds ratio (95% CI)^a^
Confronted/ reported someone	No	771 (45.6)	847 (54.4)	Reference	1804	Reference
	Yes	92 (19.8)	373 (80.2)	3.40 (2.65 to 4.37)**		2.90 (2.22 to 3.79)**
Been confronted/ reported by someone	No	745 (42.7)	1001 (57.3)	Reference	1802	Reference
	Yes	53 (19.4)	220 (80.6)	3.09 (2.26 to 4.23)**		2.59 (1.84 to 3.65)**
Other people have felt angry at me, because of my behaviour during COVID-19 pandemic	No	756 (44.8)	932 (55.2)	Reference	1704	Reference
	Yes	23 (10.6)	195 (89.4)	6.88 (4.42 to 10.71)**		5.59 (3.48 to 8.98)**
Worn a face covering in last two weeks	No	267 (48.2)	287 (51.8)	Reference	1824	Reference
	Yes	538 (36.1)	951 (63.9)	1.64 (1.35 to 2.00)**		1.68 (1.36 to 2.09)**
Stayed 2 m away from others in last two weeks	No	56 (43.8)	72 (56.3)	Reference	1828	Reference
	Yes	750 (39.1)	1168 (60.9)	1.21 (0.84 to 1.74)		1.41 (0.95 to 2.09)
Previously had COVID-19	Think not	733 (41.4)	1038 (58.6)	Reference	1814	Reference
	Think have had, or have had confirmed by a test	71 (27.8)	184 (72.2)	1.83 (1.37 to 2.45)**		1.65 (1.20 to 2.29)*
Perceived risk of COVID-19 to oneself	4-point scale, 1 = no risk at all to 4 = very high risk	N = 789, M = 2.49, SD = 0.79	N = 1236, M = 2.67, SD = 0.80	1.33 (1.19 to 1.49)**	1817	1.49 (1.32 to 1.70)**
Perceived risk of COVID-19 to people in the UK	4-point scale, 1 = no risk at all to 4 = very high risk	N = 802, M = 2.94, SD = 0.64	N = 1247, M = 3.09, SD = 0.66	1.43 (1.24 to 1.64)**	1833	1.40 (1.21 to 1.63)**
Perceived likelihood of catching COVID-19 in the next month	Range 0 (no possi bility) to 100 (definitely will)	N = 466, M = 18.59, SD = 21.65	N = 846, M = 26.04, SD = 23.71	1.01 (1.01 to 1.02)**	1192	1.01 (1.01 to 1.02)**
Subjective knowledge of COVID-19	7-point scale, 1 = very low level of knowledge to 7 = very high level of knowledge	N = 810, M = 4.70, SD = 1.35	N = 1250, M = 4.78, SD = 1.42	1.04 (0.98 to 1.11)	1840	1.09 (1.02 to 1.17)*
Identified cough, fever and loss/change of sense of smell/taste as symptoms of COVID-19	Did not identify three key symptoms	554 (39.4)	853 (60.6)	Reference	1848	Reference
	Identified three key symptoms	262 (39.5)	402 (60.5)	1.00 (0.83 to 1.20)		0.97 (0.79 to 1.20)
Stigma – best to avoid people who have recovered from COVID-19	False	416 (38.5)	665 (61.5)	Reference	1848	Reference
	True/don’t know	400 (40.4)	590 (59.6)	0.92 (0.77 to 1.10)		0.96 (0.79 to 1.17)
The government only wants us to wear face masks as a way of controlling us	Do not endorse	662 (39.5)	1013 (60.5)	Reference	1712	Reference
	Endorse	77 (31.8)	165 (68.2)	1.40 (1.05 to 1.87)*		1.18 (0.86 to 1.63)
Trust UK government to control the spread of COVID-19	4-point scale, 1 = not at all to 4 = a great deal	N = 807, M = 2.51, SD = 0.85	N = 1247, M = 2.38, SD = 0.91	0.84 (0.76 to 0.93)**	1835	0.83 (0.74 to 0.93)**
Relaxation of measures	Too quickly	366 (33.7)	720 (66.3)	1.73 (1.41 to 2.11)**	1515	1.87 (1.49 to 2.35)**
	About the right pace	286 (46.7)	326 (53.5)	Reference		Reference
	Too slowly	114 (40.9)	165 (59.1)	1.27 (0.95 to 1.69)	805	1.00 (0.72 to 1.38)
Worry about restrictions being lifted	7-point scale, 1 = not worried to 7 = very worried	N = 806, M = 4.09, SD = 1.68	N = 1234, M = 4.62, SD = 1.69	1.20 (1.14 to 1.27)**	1826	1.23 (1.16 to 1.30)**
Feeling more anxious and depressed than normal	No	514 (48.4)	549 (51.6)	Reference	1808	Reference
	Yes	284 (29.5)	679 (70.5)	2.24 (1.86 to 2.69)**		1.93 (1.58 to 2.37)**
Stress of COVID-19	7-point scale, 1 = not stressful to 7 = stressful	N = 811, M = 4.24, SD = 1.62	N = 1251, M = 4.89, SD = 1.59	1.28 (1.21 to 1.35)**	1842	1.27 (1.19 to 1.35)**
Information source about COVID-19 – TV and radio broadcasters	4-point scale, 1 = nothing at all to 4 = a great deal	N = 811, M = 3.16, SD = 0.82	N = 1249, M = 3.18, SD = 0.81	1.02 (0.92 to 1.14)	1838	1.06 (0.94 to 1.20)
Information source about COVID-19 – newspapers and magazines	4-point scale, 1 = nothing at all to 4 = a great deal	N = 808, M = 2.45, SD = 1.01	N = 1242, M = 2.61, SD = 1.00	1.17 (1.07 to 1.28)**	1830	1.15 (1.04 to 1.27)*
Information source about COVID-19 – YouTube	4-point scale, 1 = nothing at all to 4 = a great deal	N = 796, M = 1.44, SD = 0.79	N = 1229, M = 1.71, SD = 0.99	1.39 (1.25 to 1.54)**	1810	1.26 (1.11 to 1.43)**
Information source about COVID-19 – Facebook	4-point scale, 1 = nothing at all to 4 = a great deal	N = 800, M = 1.62, SD = 0.85	N = 1235, M = 1.92, SD = 0.96	1.45 (1.31 to 1.61)**	1821	1.30 (1.15 to 1.46)**
Information source about COVID-19 – WhatsApp	4-point scale, 1 = nothing at all to 4 = a great deal	N = 798, M = 1.33, SD = 0.70	N = 1219, M = 1.57, SD = 0.90	1.45 (1.29 to 1.64)**	1800	1.29 (1.12 to 1.48)**
Information source about COVID-19 – Twitter	4-point scale, 1 = nothing at all to 4 = a great deal	N = 784, M = 1.41, SD = 0.75	N = 1212, M = 1.69, SD = 0.96	1.46 (1.30 to 1.63)**	1782	1.29 (1.13 to 1.46)**
Information source about COVID-19 – family or friends	4-point scale, 1 = nothing at all to 4 = a great deal	N = 812, M = 2.32, SD = 0.82	N = 1247, M = 2.49, SD = 0.86	1.27 (1.14 to 1.41)**	1837	1.11 (0.98 to 1.25)

For non-binary independent variables, the presented odds ratios and adjusted odds ratios relate to a one-unit increase in the factor scale.

a Adjusting for gender, age, presence of dependent child in the household, employment status, highest educational or professional qualification, total annual household income, socioeconomic grade, household size, marital status, ethnicity and region.

* *p* ≤ 0.05.

** *p* ≤ 0.001.

### Confronting or reporting someone

Five hundred participants (22.4%, 95% confidence interval: 20.6–24.1) stated that they had confronted or reported someone regarding protective measures for COVID-19.

People who were: aged 16–24 years (adjusted odd ratios for different age groups: 0.49, 95% confidence interval 0.34 to 0.70 [25–34 years] to 0.24, 95% confidence interval 0.16 to 0.36 [45–54 years]); working (not working adjusted odd ratio 0.61, 95% confidence interval 0.47 to 0.79); and who reported being more likely to experience significant financial difficulty as a result of COVID-19 (adjusted odd ratio 1.44, 95% confidence interval 1.30 to 1.59); were more likely to have confronted or reported someone (full results in supplementary materials). Also associated with confrontation were: being confronted or reported (adjusted odd ratio 8.05, 95% confidence interval 5.96 to 10.88); having arguments, falling out or feeling angry with others (adjusted odd ratio 2.92, 95% confidence interval 2.24 to 3.82); thinking that other people had felt angry at you (adjusted odd ratio 3.69, 95% confidence interval 2.68 to 5.09); thinking that you had previously had COVID-19 (adjusted odd ratio 2.70, 95% confidence interval 2.00 to 3.66); endorsing a conspiracy theory about face masks (adjusted odd ratio 2.40, 95% confidence interval 1.83 to 3.15); greater use of WhatsApp (adjusted odd ratio 1.58, 95% 1.38 to 1.81), YouTube (adjusted odd ratio 1.41, 95% confidence interval 1.24 to 1.60), Facebook (adjusted odd ratio 1.36, 95% 1.20 to 1.53), and Twitter (adjusted odd ratio 1.36, 95% confidence interval 1.19 to 1.55) as information sources about COVID-19; and greater perceived risk of COVID-19 (to oneself [adjusted odd ratio 1.31, 95% confidence interval 1.14 to 1.50] and people in the UK [adjusted odd ratio 1.37, 95% confidence interval 1.16 to 1.63]); (see supplementary materials for full results).

### Having been confronted or reported by someone

A total of 304 people reported having been confronted by someone because of COVID-19 measures (13.6%, 95% confidence interval: 12.2 to 15.0).

Having been confronted was associated with younger age (adjusted odd ratios for different age groups: 0.27, 95% confidence interval 0.17 to 0.42 [35–44 years] to 0.13, 95% confidence interval 0.08 to 0.21 [55 years and over]), working (not working: adjusted odds ratio 0.57, 95% confidence interval 0.41 to 0.79), not having stayed 2 metres away from others (stayed 2 metres from others: adjusted odds ratio 0.46, 95% confidence ratio 0.29 to 0.74), and experiencing greater likelihood of significant financial difficulty from COVID-19 (adjusted odds ratio 1.50, 95% confidence interval 1.32 to 1.69; full results in supplementary materials).

Being confronted was associated with thinking that other people had felt angry at you (adjusted odds ratio 4.98, 95% confidence interval: 3.43 to 7.23); having arguments, falling out or feeling angry with others (adjusted odds ratio 2.66, 95% confidence interval: 1.87 to 3.76); and greater trust in the UK Government (F(14,1822) = 5.19, *p* < 0.001; see supplementary materials for full results).

### Discussion

The easing of restrictions around COVID-19 have caused, or revealed, tension within the population. Over half of participants reported having had arguments, feeling angry or having fallen out with others because of COVID-19. Over one in five reported having been confronted or reported to authorities. There is little research investigating normative rates of anger in the general population, with most research focusing on forensic or clinical populations. While different to anger, weekly tracker surveys conducted before the pandemic indicated that between 30% and 40% of the UK general population reported being frustrated.^20^ Further evidence suggests that 8% of the general population report inappropriate or intense anger.^21^ Unsurprisingly, anger and conflict were strongly associated with each other.

Anger was associated with lower levels of trust in the UK Government to control the spread of COVID-19, thinking that measures were being relaxed too quickly, and greater worry about restrictions being lifted. Those who are worried about the speed with which restrictions are being lifted may perceive a greater risk from COVID-19. In the days following the announcement of restrictions being eased (opening of restaurants, hairdressers and other venues), polling indicated that support for easing restrictions fell, while worry increased.^22^ We found that greater perceived risk of COVID-19 was associated with anger and conflict. Easing restrictions at a slower rate, and setting out (and sticking to) clear plans with unambiguous, measurable targets that must be achieved before easing restrictions may help increase trust in the Government, decrease worry about easing restrictions and decrease community tension.^23^

Thinking you have had COVID-19 was associated with confrontation. Those who have not had COVID-19, or who perceive a low risk of catching it, may not think the risk or necessity to adopt protective behaviours is personally relevant to themselves. As emotional arousal is associated with greater aggression,^24^ greater perceived personal relevance of the threat of COVID-19 may underlie anger and tension. Evidence suggests that people who think they have had COVID-19 are less likely to adhere to personal protective measures which prevent the spread of the virus.^19^ We found that that people who reported not adhering to physical distancing measures in the last two weeks were more likely to have been involved in confrontation. Therefore, people who reported having had COVID-19 may have been more likely to be involved in conflict due to non-adherence to personal protective behaviours.

Greater reliance on social media as an information source about COVID-19 was associated with anger and confrontation. These associations remained significant even when controlling for sociodemographic characteristics. Nowadays, an increasing proportion of the UK population uses social media for news, despite it having the poorest ratings for accuracy and trustworthiness.^25^ Misinformation and conspiracy theories about COVID-19 have spread quickly via social media and were associated with decreased adherence to Government guidelines.^12^ While endorsement of a conspiracy theory about face coverings was not associated with anger in this study, it was strongly associated with having confronted someone. Debunking misinformation and conspiracy theories may help decrease community tension.

Anger and conflict were associated with younger age and increased likelihood of facing significant financial difficulties because of the disruption from COVID-19. While our results may also reflect general findings that older adults outwardly display anger less often than younger adults,^26^ opportunity for anger and confrontation is greater in those who go out more. Younger people and those in low-income households were less likely to be able to work from home during the lockdown.^27^ These groups, and black and minority ethnic groups, have been disproportionately negatively affected by the COVID-19 pandemic.^27–29^ In our sample, black and minority ethnicity was associated with anger and confrontation in unadjusted analyses, but there was no evidence for an association when adjusting for other sociodemographic characteristics. Inequalities within the population in emergencies can lead to tension.^4,15^ Our results indicate that those most affected by the official response to the COVID-19 pandemic may also be those who experience more anger and conflict.^26^ Increasing support to reduce these inequalities may decrease community tension.

Emergencies can have a negative, lasting impact on population mental health. Psychological disorders were more prevalent in the UK population during lockdown than in the pre-pandemic period, in particular in younger age groups, those facing greater financial hardship and those who felt a loss of sense of community with people in their neighbourhood.^7,8^ Our study found that feeling more anxious and depressed than usual, and increased perceived stress of COVID-19 were associated with anger and conflict. Taken together, results suggest that providing support to those experiencing distress may also decrease community tension.

This study has several limitations. First, while quota sampling was used to ensure the sample was demographically representative of the UK general population, we cannot be sure that the views and experiences of survey respondents are representative of those of the population.^30^ However we assume, following the principles set out by Kohler,^16^ that the associations between variables within our sample follow the same pattern as those within the general population. Second, due to the cross-sectional nature of the study, we cannot be certain about the direction of causality. Third, some multivariable regressions had high percentages of missing data, and results should be taken with caution. Fourth, we did not use a validated measure of psychological distress. Fifth, because Government guidelines on face coverings differed between countries in the UK, we were unable to tell whether people were adhering to guidance.

Although exact percentages of the population reporting anger and having experienced confrontation should be taken with caution, there is good evidence that a significant proportion has experienced anger and confrontation as restrictions relating to COVID-19 have started to ease. While lockdown measures were in force, there was little social interaction. Easing of restrictions creates more opportunity for anger and confrontation, and highlights differences in risk perception within the community. Personal protective measures currently in place do not afford anonymity, providing visible evidence as to who is adhering, and who is not, to recommended or mandated public health measures. Findings from this study reflect those from previous emergencies in which corrosive communities have emerged and suggest that we may be moving from therapeutic communities towards ones characterised more by corrosion.

### Supplemental Material

sj-pdf-1-jrs-10.1177_0141076820962068 - Supplemental material for Anger and confrontation during the COVID-19 pandemic: a national cross-sectional survey in the UK Click here for additional data file. Supplemental material, sj-pdf-1-jrs-10.1177_0141076820962068 for Anger and confrontation during the COVID-19 pandemic: a national cross-sectional survey in the UK by Louise E Smith, Bobby Duffy, Vivienne Moxham-Hall, Lucy Strang, Simon Wessely and G James Rubin in Journal of the Royal Society of Medicine

## Declarations

**Ethical approval:** Ethical approval for this study was granted by the King’s College London Research Ethics Committee (reference: MRA-19/20-18251).

**Guarantor:** LES.

**Contributorship:** The study was conceptualised by LES, BD, SW and GJR. All authors were involved in survey design, and LS and VMH organised data collection. LES completed data analyses and wrote the first draft of the manuscript. All authors contributed and approved the final draft of the manuscript.

## Note

a. At the time of data collection wearing a face covering was mandatory on public transport but was not mandatory in shops in England and Northern Ireland. An announcement had been made indicating that mandatory face coverings would be made mandatory in shops in England, but had not yet come into effect. In Scotland, face coverings were mandatory both on public transport and in shops. In Wales, face coverings were not mandatory on public transport or in shops.
