# Supplementary material for: Anger and confrontation during the COVID-19 pandemic: a national cross-sectional survey in the UK
Source: J R Soc Med. 2020 Oct 28;114(2):77–90. doi: 10.1177/0141076820962068 (PMC7876655; doi:10.1177/0141076820962068)
Supplement: sj-pdf-1-jrs-10.1177_0141076820962068 - Supplemental material for Anger and confrontation during the COVID-19 pandemic: a national cross-sectional survey in the UK [file sj-pdf-1-jrs-10.1177_0141076820962068.pdf]

Supplementary materials. Full survey questions.

Factor	Question	Response scale
Anger	For each of the following, please say whether it does or does not apply to you?<br>- I have had arguments with friends or family members about how to behave during the coronavirus pandemic<br>- I have felt angry with other people I know because of how they are behaving in relation to the coronavirus pandemic<br>- I think other people I know have felt angry at me, because of how I am behaving in relation to the coronavirus pandemic<br>- I am no longer speaking to a friend or family member because of disagreements about the coronavirus pandemic.	• Applies to me<br>• Does not apply to me<br>• Don't know
Confrontation	Have you personally done or experienced any of the following in the past month? Please answer honestly, all answers to this survey are anonymous.<br>- Confronted someone for not wearing a face mask<br>- Reported someone to the authorities for not wearing a face mask<br>- Been confronted by someone because you were not wearing a face mask<br>- Been reported to the authorities by someone because you were not wearing a face mask<br>- Confronted someone for not staying a sufficient distance away from others or for being in too large a group<br>- Been confronted by someone because you were not staying a sufficient distance away from others or for being in too large a group<br>- Confronted someone for following the recommended measures too carefully<br>- Been confronted by someone for following the recommended measures too carefully	• Yes<br>• No<br>• Don't know
Self-reported protective behaviours	Which of the following measures, if any, have you personally taken over the past two weeks because you believe they may prevent the spread of coronavirus?<br>Please only choose options that you have started doing or are doing more because of the coronavirus.<br>- Staying 2 metres away from other people when outside your home<br>- Wearing a face mask or covering	• Yes<br>• No<br>• Don't know
Beliefs and knowledge about COVID-19	Do you have, or have you had, the coronavirus?	• Yes, confirmed by a test<br>• Yes, I'm sure I / they have, but not confirmed by a test<br>• I'm not sure, but I think so<br>• I'm not sure, but I don't think so<br>• No

	• Don't know<br>• Prefer not to say
To what extent, if at all, do you think the coronavirus poses a risk to…?<br>- Yourself personally<br>- People in the UK	• A very high risk<br>• A fairly high risk<br>• Not a very high risk<br>• No risk at all<br>• Don't know
What do you think your personal chance of catching the coronavirus is, or if you have already had it, of catching it again…<br>Please note 0 means there is no possibility you will, and 100 means you definitely will.<br>- …in the next month	Write in numeric 0-100
Are the following statements true or false?<br>- It's best to avoid people who have recovered from coronavirus.	• True<br>• False<br>• Don't know
How would you rate your knowledge level about the coronavirus? Please answer on a scale from 1 to 7, where 1 is very high level of knowledge and 7 is very low level of knowledge.	1. Very high level of knowledge<br>2.<br>3.<br>4.<br>5.<br>6.<br>7. Very low level of knowledge<br>8. Don't know
Can you tell us what you think the most common symptoms of coronavirus are?<br>Please select up to 3.	• Cough<br>• High temperature / fever<br>• Shortness of breath / difficulties breathing<br>• Runny or blocked nose<br>• Aches and pains<br>• Chest pain<br>• Chills / shivering<br>• Sore throat<br>• Diarrhoea<br>• Headache<br>• Stomach ache<br>• Feeling tired or having low energy

	Are the following statements true or false?<br>- The government only wants us to wear face masks as a way of controlling us.	• Loss of sense of smell or taste<br>• None of these<br>• Don’t know<br>• True<br>• False<br>• Don’t know
Trust in UK Government and COVID-19 measures	To what extent, if at all, do you trust…<br>- the UK Government to control the spread of the coronavirus?	• A great deal<br>• A fair amount<br>• Not very much<br>• Not at all<br>• Don’t know
	And do you think the UK Government is relaxing the measures to control coronavirus too quickly, too slowly, or at about the right pace?<br><br>Which of these is closest to your view…?	• Too quickly<br>• Too slowly<br>• About the right pace<br>• Don’t know<br>1. I am very worried about the restrictions being lifted<br>2.<br>3.<br>4.<br>5.<br>6.<br>7. I am not worried about the restrictions being lifted<br>8. Don’t know
Psychological factors	Have you personally done or experienced any of the following in the past month? Please answer honestly, all answers to this survey are anonymous.<br>- Felt more anxious and depressed than normal<br>Please choose one option for the following questions, on a scale of 1 to 7.<br>The coronavirus to me is...	• Yes<br>• No<br>• Don’t know<br>1. Stressful<br>2.<br>3.<br>4.<br>5.<br>6.<br>7. Not stressful<br>8. Don’t know

Information sources	Please tell us how much of what you know about coronavirus, if anything, comes from…<br>- TV and radio broadcasters (including through their websites and online)<br>- Newspapers and magazines (including through their websites and online)<br>- YouTube<br>- Facebook<br>- WhatsApp<br>- Twitter<br>- Family or friends	• A great deal<br>• A fair amount<br>• Not very much<br>• Nothing at all<br>• Don’t know
Socio-demographic characteristics	And how likely or unlikely do you think it is that you will face significant financial difficulties such as not being able to pay for your housing or essential items as a result of the disruption from coronavirus?	• Certain<br>• Very likely<br>• Fairly likely<br>• Not very likely<br>• Not at all likely<br>• I already am<br>• Don’t know

Supplementary materials. Recoding of variables.

We created separate binary variables indicating whether participants had been confronted or reported for any reason, or if they had been confronted or reported someone else for any reason.

We created a single binary variable indicating whether participants had had arguments with, or were no longer speaking to, friends or family members because of the COVID-19 pandemic.

For the likelihood of facing significant financial difficulties variable, we grouped answers of “certain” and “I already am”.

We coded participants into two groups: thinking they had previously had COVID-19, or not.

We categorised people as knowing the symptoms of COVID-19 if they correctly identified cough, high temperature / fever and loss or change of sense of smell or taste as symptoms of COVID-19.

To investigate perceived speed at which restrictions were being lifted, we created two separate dummy variables, comparing “too quickly” and “too slowly” with “about the right pace”.

We recoded endorsement of a face covering conspiracy theory into a binary variable, grouping answers of “true” and “don’t know” together (versus “false”).

Unless otherwise specified, we coded answers of “don’t know” or “prefer not to say” as missing.

Supplementary materials. Full tables for associations between having been confronted or reported.

Table depicting associations between having been confronted or reported and anger, stigma and feeling more anxious and depressed than normal.

Having been confronted / reported	Had arguments or fallen out / felt angry with others because of the COVID-19 pandemic				
	Univariable analyses			Multivariable analyses	
	No n=798	Yes n=1221	Odds ratio (95% CI)	Total n	Adjusted odds ratio (95% CI)†
Has not been confronted	745 (42.7)	1001 (57.3)	Reference	1771	Reference
Has been confronted	53 (19.4)	220 (80.6)	3.09 (2.26 to 4.23)**		2.66 (1.87 to 3.76)**
	Other people have felt angry at me, because of my behaviour during COVID-19 pandemic				
	Univariable analyses			Multivariable analyses	
	No n=1714	Yes n=219	Odds ratio (95% CI)	Total n	Adjusted odds ratio (95% CI)†
Has not been confronted	1540 (92.5)	124 (7.5)	Reference	1687	Reference
Has been confronted	174 (64.7)	95 (35.3)	6.78 (4.97 to 9.24)**		4.98 (3.43 to 7.23)**
	Stigma – best to avoid people who have recovered from COVID-19				
	Univariable analyses			Multivariable analyses	
	False n=1108	True / don't know n=1022	Odds ratio (95% CI)	Total n	Adjusted odds ratio (95% CI)†
Has not been confronted	968 (53.0)	858 (47.0)	Reference	1852	Reference
Has been confronted	140 (46.1)	164 (53.9)	1.32 (1.04 to 1.69)*		1.36 (1.03 to 1.80)*
	Feeling more anxious and depressed than normal				
	Univariable analyses			Multivariable analyses	
	No n=1099	Yes n=992	Odds ratio (95% CI)	Total n	Adjusted odds ratio (95% CI)†
Has not been confronted	967 (54.3)	822 (45.7)	Reference	1822	Reference
Has been confronted	123 (42.0)	170 (58.0)	1.64 (1.28 to 2.11)**		1.54 (1.16 to 2.06)*

*$p \leq .05$
**$p \leq .001$
† Adjusting for gender, age, presence of dependent child in the household, employment status, highest educational or professional qualification, total annual household income, socio-economic grade, household size, marital status, ethnicity, region, wearing a mask and staying 2m away from others.

Table depicting associations between having been confronted or reported and COVID-19 stress, trust in UK Government and information sources.

Having been confronted		COVID-19 stress										
		Unadjusted analyses					Adjusted analyses†					
		Model			Regression coefficient		Model				Regression coefficient	
	7-point scale, 1=not worried to 7=very worried	*F*	Adjusted R^2	*p*	β	*p*	*df*	*F*	Adjusted R^2	*p*	β	*p*
Has not been confronted	N=1820, M=4.63, SD=1.63											
Has been confronted	N=302, M=4.64, SD=1.64	0.03	.000	.87	.00	.87	14,1831	13.48	.087	<.001**	.02	.45
		Trust UK government to control the spread of COVID-19										
		Unadjusted analyses					Adjusted analyses†					
		Model			Regression coefficient		Model				Regression coefficient	
	4-point scale, 1=not at all to 4=a great deal	*F*	Adjusted R^2	*p*	β	*p*	*df*	*F*	Adjusted R^2	*p*	β	*p*
Has not been confronted	N=1811, M=2.41, SD=0.87											
Has been confronted	N=301, M=2.63, SD=0.96	16.43	.007	<.001**	.09	<.001**	14,1822	5.19	.031	<.001**	.10	<.001**

*$p \leq .05$
**$p \leq .001$
† Adjusting for gender, age, presence of dependent child in the household, employment status, highest educational or professional qualification, total annual household income, socio-economic grade, household size, marital status, ethnicity, region, wearing a mask and staying 2m away from others.

Table depicting associations between socio-demographic characteristics of participants and having confronted or reported someone.

		Univariable analyses			Multivariable analyses	
Participant characteristics	Level	Not confronted / reported anyone n=1637	Confronted / reported someone n=500	Odds ratio (95% CI)	Total n	Adjusted odds ratio (95% CI)†
Gender	Male	783 (74.1)	273 (25.9)	Reference	1899	Reference
	Female	835 (79.0)	222 (21.0)	0.76 (0.62 to 0.93)*		0.72 (0.58 to 0.90)*
Age	16 to 24 years	188 (60.8)	121 (39.2)	Reference	1899	Reference
	25 to 34 years	277 (71.4)	111 (28.6)	0.62 (0.45 to 0.86)*		0.49 (0.34 to 0.70)**
	35 to 44 years	281 (78.3)	78 (21.7)	0.43 (0.31 to 0.61)**		0.34 (0.23 to 0.50)**
	45 to 54 years	352 (82.6)	74 (17.4)	0.33 (0.23 to 0.46)**		0.24 (0.16 to 0.36)**
	55 years and over	539 (82.3)	116 (17.7)	0.33 (0.25 to 0.45)**		0.25 (0.17 to 0.37)**
Child in the household	None	1221 (78.0)	344 (22.0)	Reference	1899	Reference
	Child present	416 (72.7)	156 (27.3)	1.33 (1.07 to 1.66)*		1.18 (0.86 to 1.62)
Employment status	Working	994 (73.5)	359 (26.5)	Reference	1899	Reference
	Not working	643 (82.0)	141 (18.0)	0.61 (0.49 to 0.76)**		0.61 (0.47 to 0.79)**
Highest educational or professional qualification	Degree or higher (Bachelors, Masters, PhD)	697 (76.8)	211 (23.2)	Reference	1899	Reference
	GCSE/vocational/A-level/No formal qualifications	940 (76.5)	289 (23.5)	1.02 (0.83 to 1.24)		1.20 (0.94 to 1.52)
Total annual household income	Up to £34,999	903 (77.2)	267 (22.8)	Reference	1899	Reference
	£35,000 or over	574 (74.5)	196 (25.5)	1.15 (0.93 to 1.43)		0.91 (0.71 to 1.17)
Socio-economic grade	ABC1	1051 (76.0)	332 (24.0)	Reference	1899	Reference
	C2DE	586 (77.7)	168 (22.3)	0.91 (0.73 to 1.12)		0.97 (0.76 to 1.24)
Household size	One	350 (77.4)	102 (22.6)	Reference	1899	Reference
	Two	593 (78.3)	164 (21.7)	0.95 (0.72 to 1.26)		0.72 (0.49 to 1.06)
	Three or more	694 (74.8)	234 (25.2)	1.16 (0.89 to 1.51)		0.62 (0.41 to 0.92)*
Marital status	Married/living as married	908 (76.7)	276 (23.3)	Reference	1899	Reference
	Single/separated/divorced/widowed	729 (76.5)	224 (23.5)	1.01 (0.83 to 1.24)		0.71 (0.53 to 0.96)
Ethnicity	White	1477 (77.5)	430 (22.5)	Reference	1899	Reference
	Black and minority ethnicity	137 (68.5)	63 (31.5)	1.58 (1.15 to 2.17)*		1.03 (0.71 to 1.49)
Region	North	379 (76.3)	118 (23.7)	Reference	1899	Reference
	Midlands	416 (77.9)	118 (22.1)	0.91 (0.68 to 1.22)		0.88 (0.64 to 1.22)
	South	378 (79.6)	97 (20.4)	0.82 (0.61 to 1.12)		0.85 (0.61 to 1.17)
	London	208 (71.7)	82 (28.3)	1.27 (0.91 to 1.76)		1.2 (0.82 to 1.73)
	Wales	69 (68.3)	32 (31.7)	1.49 (0.93 to 2.38)		1.56 (0.94 to 2.59)
	Scotland	143 (77.3)	42 (22.7)	0.94 (0.63 to 1.41)		0.89 (0.57 to 1.38)
	Northern Ireland	44 (80.0)	11 (22.0)	0.80 (0.40 to 1.60)		0.72 (0.33 to 1.56)

Significant financial difficulty	5-point scale, 1=not at all likely to 5=certain/already am	N=1437, M=2.21, SD=1.10	N=459, M=2.77, SD=1.35	1.46 (1.34 to 1.59)**	1713	1.44 (1.30 to 1.59)**

For non-binary independent variables, the presented odds ratios and adjusted odds ratios relate to a one-unit increase in the factor scale.

*$p \leq .05$

**$p \leq .001$

† Adjusting for gender, age, presence of dependent child in the household, employment status, highest educational or professional qualification, total annual household income, socio-economic grade, household size, marital status, ethnicity, and region.

Table depicting associations between anger, protective behaviours, beliefs and knowledge about COVID-19, trust in UK Government and COVID-19 measures, psychological factors, information sources and having confronted or reported someone.

		Univariable analyses			Multivariable analyses	
Factor	Level	Not confronted / reported anyone n=1637	Confronted / reported someone n=500	Odds ratio (95% CI)	Total n	Adjusted odds ratio (95% CI)†
Been confronted / reported	No<br>Yes	1514 (83.6)<br>103 (35.9)	296 (16.4)<br>184 (64.1)	Reference<br>9.14 (6.97 to 11.99)**	1863	Reference<br>8.05 (5.96 to 10.88)**
Had arguments or fallen out / felt angry with others because of the COVID-19 pandemic	No<br>Yes	711 (88.5)<br>847 (69.4)	92 (11.5)<br>373 (30.6)	Reference<br>3.40 (2.65 to 4.37)**	1804	Reference<br>2.92 (2.24 to 3.82)**
Other people have felt angry at me, because of my behaviour during COVID-19 pandemic	No<br>Yes	1380 (80.7)<br>109 (47.4)	330 (19.3)<br>121 (52.6)	Reference<br>4.64 (3.49 to 6.18)**	1727	Reference<br>3.69 (2.68 to 5.09)**
Worn a face covering in last two weeks	No<br>Yes	463 (80.9)<br>1153 (75.1)	109 (19.1)<br>383 (24.9)	Reference<br>1.41 (1.11 to 1.79)*	1874	Reference<br>1.32 (1.01 to 1.71)*
Stayed 2m away from others in last two weeks	No<br>Yes	87 (65.9)<br>1529 (77.3)	45 (34.1)<br>450 (22.7)	Reference<br>0.57 (0.39 to 0.83)	1878	Reference<br>0.66 (0.44 to 0.99)*
Previously had COVID-19	Think not<br>Think have had, or have had confirmed by a test	1452 (79.7)<br>149 (55.8)	369 (20.3)<br>118 (44.2)	Reference<br>3.12 (2.39 to 4.07)**	1861	Reference<br>2.70 (2.00 to 3.66)**
Perceived risk of COVID-19 to oneself	4-point scale, 1=no risk at all to 4=very high risk	N=1593, M=2.57, SD=0.79	N=489, M=2.69, SD=0.82	1.19 (1.05 to 1.35)*	1861	1.31 (1.14 to 1.50)**
Perceived risk of COVID-19 to people in the UK	4-point scale, 1=no risk at all to 4=very high risk	N=1614, M=2.99, SD=0.64	N=496, M=3.14, SD=0.69	1.40 (1.20 to 1.64)**	1880	1.37 (1.16 to 1.63)**
Perceived likelihood of catching COVID in the next month	Range 0 (no possibility) to 100 (definitely will)	N=1007, M=22.82, SD=22.97	N=340, M=26.04, SD=24.58	1.01 (1.00 to 1.01)*	1220	1.01 (1.00 to 1.01)
Subjective knowledge of COVID-19	7-point scale, 1=very low level of knowledge to 7=very high level of knowledge	N=1626, M=4.75, SD=1.35	N=496, M=4.66, SD=1.56	0.96 (0.89 to 1.03)	1889	0.98 (0.91 to 1.06)
Identified cough, fever, and loss/change of sense of smell/taste as symptoms of COVID-19	Did not identify three key symptoms<br>Identified three key symptoms	1083 (74.5)<br>554 (81.0)	370 (25.5)<br>130 (19.0)	Reference<br>0.69 (0.55 to 0.86)**	1899	Reference<br>0.73 (0.57 to 0.94)*
Stigma – best to avoid people who have recovered from COVID-19	False<br>True / don't know	876 (78.7)<br>761 (64.3)	237 (21.3)<br>263 (25.7)	Reference<br>1.28 (1.05 to 1.56)*	1899	Reference<br>1.31 (1.05 to 1.63)*
The government only wants us to wear face masks as a way of controlling us	Do not endorse<br>Endorse	1341 (78.7)<br>153 (59.3)	364 (21.3)<br>105 (40.7)	Reference<br>2.53 (1.92 to 3.33)**	1899	Reference<br>2.40 (1.83 to 3.15)**

Trust UK government to control the spread of COVID-19	4-point scale, 1=not at all to 4=a great deal	N=1622, M=2.42, SD=0.86	N=497, M=2.48, SD=0.96	1.08 (0.97 to 1.21)	1884	1.06 (0.94 to 1.20)
Relaxation of measures	Too quickly	852 (76.3)	264 (23.7)	1.15 (0.91 to 1.45)	1550	1.22 (0.94 to 1.58)
	About the right pace	497 (78.8)	134 (21.2)	Reference		Reference
	Too slowly	206 (70.5)	86 (29.5)	1.55 (1.13 to 2.12)*	830	1.23 (0.86 to 1.75)
Worry about restrictions being lifted	7-point scale, 1=not worried to 7=very worried	N=1606, M=4.39, SD=1.72	N=494, M=4.44, SD=1.68	1.02 (0.96 to 1.08)	1871	1.03 (0.96 to 1.10)
Feeling more anxious and depressed than normal	No	887 (80.5)	215 (19.5)	Reference	1864	Reference
	Yes	724 (72.6)	273 (27.4)	1.56 (1.27 to 1.91)**		1.42 (1.13 to 1.79)*
Stress of COVID-19	7-point scale, 1=not stressful to 7= stressful	N=1630, M=4.58, SD=1.63	N=498, M=4.80, SD=1.62	1.09 (1.02 to 1.16)*	1891	1.10 (1.03 to 1.18)*
Information source about COVID-19 – TV and radio broadcasters	4-point scale, 1=nothing at all to 4=a great deal	N=1628, M=3.16, SD=0.82	N=496, M=3.18, SD=0.82	1.02 (0.90 to 1.15)	1888	1.02 (0.89 to 1.17)
Information source about COVID-19 – newspapers and magazines	4-point scale, 1=nothing at all to 4=a great deal	N=1620, M=2.49, SD=1.01	N=496, M=2.73, SD=0.99	1.28 (1.15 to 1.41)**	1882	1.20 (1.07 to 1.34)*
Information source about COVID-19 – YouTube	4-point scale, 1=nothing at all to 4=a great deal	N=1597, M=1.51, SD=0.84	N=493, M=1.97, SD=1.11	1.61 (1.46 to 1.78)**	1860	1.41 (1.24 to 1.60)**
Information source about COVID-19 – Facebook	4-point scale, 1=nothing at all to 4=a great deal	N=1605, M=1.72, SD=0.88	N=497, M=2.10, SD=1.03	1.53 (1.38 to 1.70)**	1873	1.36 (1.20 to 1.53)**
Information source about COVID-19 – WhatsApp	4-point scale, 1=nothing at all to 4=a great deal	N=1597, M=1.39, SD=0.74	N=485, M=1.86, SD=1.06	1.78 (1.59 to 1.98)**	1850	1.58 (1.38 to 1.81)**
Information source about COVID-19 – Twitter	4-point scale, 1=nothing at all to 4=a great deal	N=1581, M=1.49, SD=0.82	N=474, M=1.89, SD=1.05	1.58 (1.42 to 1.76)**	1827	1.36 (1.19 to 1.55)**
Information source about COVID-19 – family or friends	4-point scale, 1=nothing at all to 4=a great deal	N=1625, M=2.37, SD=0.81	N=495, M=2.63, SD=0.93	1.43 (1.27 to 1.61)**	1884	1.23 (1.08 to 1.41)*

For non-binary independent variables, the presented odds ratios and adjusted odds ratios relate to a one-unit increase in the factor scale.

*$p \leq .05$

**$p \leq .001$

† Adjusting for gender, age, presence of dependent child in the household, employment status, highest educational or professional qualification, total annual household income, socio-economic grade, household size, marital status, ethnicity, and region.

Table depicting associations between socio-demographic characteristics of participants and having been confronted or reported.

		Univariable analyses			Multivariable analyses	
Participant characteristics	Level	Not been confronted /reported n=1826	Been confronted /reported n=304	Odds ratio (95% CI)	Total n	Adjusted odds ratio (95% CI)†
Gender	Male	833 (83.9)	170 (16.1)	Reference	1852	Reference
	Female	922 (87.6)	13 (12.4)	0.74 (0.58 to 0.94)*		0.74 (0.56 to 0.98)*
Age	16 to 24 years	211 (69.2)	94 (30.8)	Reference	1852	Reference
	25 to 34 years	294 (76.2)	92 (23.8)	0.70 (0.50 to 0.98)*		0.53 (0.36 to 0.80)*
	35 to 44 years	312 (87.6)	44 (12.4)	0.32 (0.21 to 0.47)**		0.27 (0.17 to 0.42)**
	45 to 54 years	395 (93.4)	28 (6.6)	0.16 (0.10 to 0.25)**		0.13 (0.08 to 0.22)**
	55 years and over	614 (93.0)	46 (7.0)	0.17 (0.11 to 0.25)**		0.13 (0.08 to 0.21)**
Child in the household	None	1369 (87.8)	191 (12.2)	Reference	1852	Reference
	Child present	457 (80.2)	113 (19.8)	1.77 (1.37 to 2.29)**		1.52 (1.03 to 2.26)*
Employment status	Working	114 (83.2)	225 (16.8)	Reference	1852	Reference
	Not working	712 (90.0)	79 (10.0)	0.55 (0.42 to 0.72)**		0.57 (0.41 to 0.79)**
Highest educational or professional qualification	Degree or higher (Bachelors, Masters, PhD)	765 (84.5)	140 (15.5)	Reference	1852	Reference
	GCSE/vocational/A-level/No formal qualifications	1061 (86.6)	164 (13.4)	0.84 (0.66 to 1.08)		1.03 (0.76 to 1.40)
Total annual household income	Up to £34,999	1007 (85.8)	167 (14.2)	Reference	1852	Reference
	£35,000 or over	638 (83.9)	122 (16.1)	1.15 (0.89 to 1.49)		0.81 (0.59 to 1.11)
Socio-economic grade	ABC1	1174 (85.2)	204 (14.8)	Reference	1852	Reference
	C2DE	652 (86.7)	100 (13.3)	0.88 (0.68 to 1.14)		1.08 (0.79 to 1.48)
Household size	One	390 (85.7)	65 (14.3)	Reference	1852	Reference
	Two	658 (87.9)	91 (12.1)	0.83 (0.59 to 1.17)		0.58 (0.36 to 0.94)*
	Three or more	778 (84.0)	148 (16.0)	1.14 (0.83 to 1.57)		0.46 (0.28 to 0.75)*
Marital status	Married/living as married	1022 (86.4)	161 (13.6)	Reference	1852	Reference
	Separated/divorced/widowed/never married	804 (84.9)	143 (15.1)	1.13 (0.89 to 1.44)		0.65 (0.45 to 0.95)*
Ethnicity	White	1660 (87.0)	248 (13.0)	Reference	1852	Reference
	Black and minority ethnicity	140 (72.2)	54 (27.8)	2.58 (1.84 to 3.63)**		1.24 (0.82 to 1.87)
Region	North	433 (86.8)	66 (13.2)	Reference	1852	Reference
	Midlands	460 (87.6)	65 (12.4)	0.93 (0.64 to 1.34)		0.95 (0.63 to 1.44)
	South	426 (88.8)	54 (11.3)	0.83 (0.57 to 1.22)		0.85 (0.55 to 1.31)
	London	219 (76.0)	69 (24.0)	2.07 (1.42 to 3.01)**		1.97 (1.26 to 3.07)*
	Wales	85 (85.0)	15 (15.0)	1.16 (0.63 to 2.12)		1.24 (0.62 to 2.47)
	Scotland	157 (86.3)	25 (13.7)	1.04 (0.64 to 1.71)		1.17 (0.68 to 2.02)
	Northern Ireland	46 (82.1)	10 (17.9)	1.43 (0.69 to 2.96)		1.51 (0.66 to 3.44)
	No	496 (87.3)	72 (12.7)	Reference	1852	Reference

Worn a face covering in last two weeks	Yes	1308 (85.4)	224 (14.6)	1.18 (0.89 to 1.57)		1.08 (0.77 to 1.51)
Stayed 2m away from others in last two weeks	No	91 (70.0)	39 (30.0)	Reference	1852	Reference
	Yes	1712 (86.8)	260 (13.2)	0.35 (0.24 to 0.53)**		0.46 (0.29 to 0.74)**
Significant financial difficulty	5-point scale, 1=not at all likely to 5=certain/already am	N=1604, M=2.22, SD=1.13	N=285, M=2.96, SD=1.24	1.63 (1.47 to 1.80)**	1672	1.50 (1.32 to 1.69)**

For non-binary independent variables, the presented odds ratios and adjusted odds ratios relate to a one-unit increase in the factor scale.

*$p \leq .05$

**$p \leq .001$

† Adjusting for gender, age, presence of dependent child in the household, employment status, highest educational or professional qualification, total annual household income, socio-economic grade, household size, marital status, ethnicity, region, wearing a mask and staying 2m away from others.
